# Bioprocessing of Marine Chitinous Wastes for the Production of Bioactive Prodigiosin

**DOI:** 10.3390/molecules26113138

**Published:** 2021-05-24

**Authors:** Thi-Hanh Nguyen, San-Lang Wang, Dai-Nam Nguyen, Anh-Dzung Nguyen, Thi-Huyen Nguyen, Manh-Dung Doan, Van-Anh Ngo, Chien-Thang Doan, Yao-Haur Kuo, Van-Bon Nguyen

**Affiliations:** 1 Department of Science and Technology, Tay Nguyen University, Buon Ma Thuot 630000, Vietnam; nguyenhanh2208.tn@gmail.com (T.-H.N.); dainamnguyen.edu@gmail.com (D.-N.N.); dcthang@ttn.edu.vn (C.-T.D.); 2 Department of Chemistry, Tamkang University, New Taipei City 25137, Taiwan; 3 Life Science Development Center, Tamkang University, New Taipei City 25137, Taiwan; 4 Institute of Biotechnology and Environment, Tay Nguyen University, Buon Ma Thuot 630000, Vietnam; nadzung@ttn.edu.vn (A.-D.N.); nthuyen@ttn.edu.vn (T.-H.N.); dmdung@ttn.edu.vn (M.-D.D.); nvanh@ttn.edu.vn (V.-A.N.); 5 Division of Chinese Materia Medica Development, National Research Institute of Chinese Medicine, Taipei 11221, Taiwan; kuoyh@nricm.edu.tw

**Keywords:** prodigiosin, *Serratia marcescens*, shrimp shells, bioreactor, fermentation, antioxidants, anti-NO activity

## Abstract

Recently, microbial prodigiosin (PG) has received much attention due to its numerous beneficial applications. The aim of this study was to establish the bioprocessing of marine chitinous wastes (MCWs) for the cost-effective preparation of PG. Of the MCWs, demineralized shrimp shell powders (de-SSP) were found to be a potential source of carbon/nitrogen (C/N) for PG production by bacterial fermentation using *Serratia marcescens* strains. Further, PG scale-up production was investigated in a 15 L bioreactor system, and the highest yield (6200 mg/L) was achieved during fermentation using 5 L of a novel-designed culture broth that included 1.60% C/N sources (a de-SSP/casein ratio of 7/3), 0.02% K_2_SO_4,_ and 0.05% K_2_HPO_4_, with an initial pH of 6–7. Fermentation was conducted in the dark at 27.5 °C for 8.0 h. This study was the first to report on the utilization of shrimp wastes for cost-effective, large-scale (5 L/pilot) PG production with high productivity (6200 mg/L) in a short cultivation time. The combination of 0.02% K_2_SO_4_ and 0.05% K_2_HPO_4_ was also found to be a novel salt composition that significantly enhanced PG yield. The red compound was purified and confirmed as PG after analyzing its HPLC profile, mass, and UV/vis spectra. The purified PG was then tested for its bioactivities and showed effective anticancer activities, moderated antioxidant activities, and novel anti-NO effects.

## 1. Introduction

Prodigiosin (PG) is a red pigment compound that belongs to the prodiginine family. PG is a metabolite of various bacteria, such as *Serratia marcescens, Alteromonas rubra, Rugamonas rubra, Streptomyces coelicolor, Serratia rubidaea, Janthinobacterium lividum, Streptoverticillium rubrireticuli,* etc. [1]. Of these bacteria, *Serratia marcescens* has most commonly been reported to be used for PG production [2].

PG has been reported to possess various biological effects, including antibacterial, algicidal, antioxidant, immunosuppressant, anti-inflammatory, anti-Alzheimer’s, antiparasitic, and insecticidal activities [3,4,5,6,7]. This microbial pigment is also commonly used in food colorants, candles, textiles, and cosmetics, and has been recently used in novel solar cells [2,8]. Notably, PG shows high anticancer properties without causing toxicity to normal cells [2,9]. 

Numerous beneficial bioactivities of PG and its applications have led to a dramatic increase in the investigation of PG biosynthesis, and numerous studies on PG production have been published. However, in almost all previous reports, commercial nutrient mediums were used as C/N sources for fermentation, such as tryptone soy, tryptone yeast, yeast malt, glycerol [10], yeast extract [11], nutrient broth [12], glycerol-tryptone [13], peptone-glycerol [14], Luria/Bertani broth [10], and 3-[*N*-morpholino]-ethanesulfonic acid [15]. Some nontraditional media, such as crude glycerol, peanut oil, sesame seed, corn steep, cassava, coconut oil, sesame oil, peanut seed, copra seed, and the complexes of mannitol/corn steep and mannitol/cassava, have been investigated for the lower-cost production of PG [6,16,17,18,19,20]. In this study, we reported for the first time the reuse of shrimp wastes as the source of C/N for PG synthesis by bacterial fermentation. 

Shrimp shell is one of the most abundant marine chitinous materials mainly obtained from by-products of fishery processing. Shrimp shells and crab shells have been widely utilized for chitin and chitosan preparation via chemical processes [21,22,23]. However, chemical preparation results in environmental issues; thus, the use of microbial technology for the production of chitin and chitosan from chitinous waste is the current trend [24,25]. Through microbial conversion, various other bioactive materials such as proteases, chitinase, chitosanases, and oligomers of chitin and chitosan, as well as antioxidants, anticancer, and antidiabetic agents have been produced from shrimp shells [26,27,28,29,30].

In our previous report, we showed that chitin plays a key role in enhancing PG yield via *S. marcescens* fermentation, and α-chitin was found to be a more effective PG enhancing agent, compared to β-chitin and other carbon sources [31]. Squid pen powder (containing β-chitin) and crab shell powder (containing α-chitin) have been extensively studied for PG production [2,32,33]. However, no study has reported the use of shrimp shells, which have the largest amount of chitinous wastes containing α-chitin, for PG production via microbial fermentation. Thus, in this study, we established the reuse of this low-cost material for the biosynthesis of PG in a flask (a small scale) and reported PG production scale-up in a bioreactor system, its purification, and the evaluation of biological activities.

## 2. Results and Discussion

### 2.1. Reclamation of Demineralized Shrimp Shell Powders (de-SSP) as a Potential Source for Effective Production of Prodigiosin via Fermentation 

**Figure 1:**
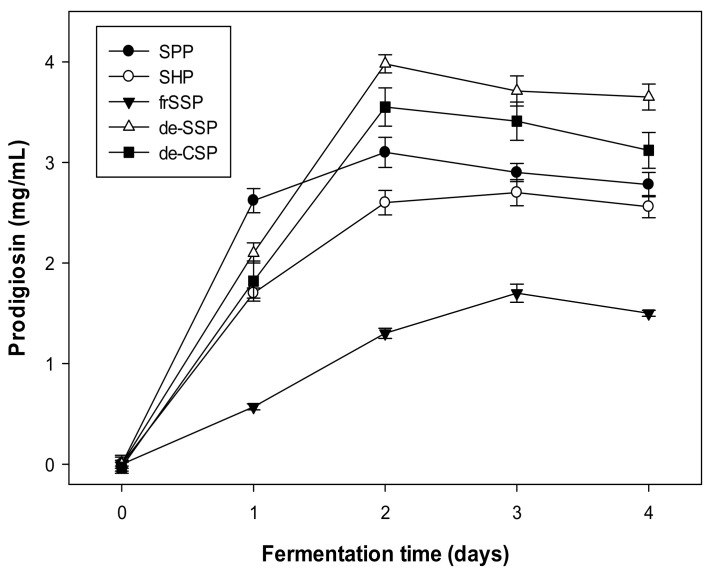
Bioproduction of PG by *S. marcescens* TNU02 by fermentation using various MCWs, such as squid pen powder (SPP), shrimp head powder (SHP), fresh shrimp shell powder (fr-SSP), demineralized crab shell powder (de-CSP), and demineralized shrimp shell powder (de-SSP), as major C/N sources with supplementary casein as a free protein at the ratio of 7.0/3.0. The C/N source (1.60%) was added to a liquid medium of 0.03% K_2_HPO_4_ and 0.05% CaSO_4_. The fermentation was performed for two days at 150 rpm (shaking speed) in the dark at 25℃. The error bars in the figures are standard errors (SE).

Various kinds of MCWs, including squid pen powder (SPP), shrimp head powder (SHP), fresh shrimp shell powder (fr-SSP), demineralized crab shell powder (de-CSP), and demineralized shrimp shell powder (de-SSP) were used for *Serratia marcescens* TUN02 fermentation and PG biosynthesis comparison. As shown in Figure 1, *S. marcescens* TUN02 produced PG at a high level on the first day of fermentation (2.62 mg/mL) in the medium containing SPP; however, the PG yield reached its highest (3.98 mg/mL) on day two in the medium containing de-SSP. Thus, de-SSP was chosen as the low-cost material for all following experiments. 

Notably, in this experiment, we found that the two shrimp shell materials gave quite different results. The fermented culture broth reached a high level of PG yield (3.98 mg/mL) using demineralized shrimp shell powder and a low PG yield (1.32 mg/mL) using fresh shrimp shells as the C/N sources for fermentation. The mineral salt content in the shrimp shells was 14% (*w*/*w*), which was too high of a concentration for microbes to grow properly, leading to a reduction in the production of microbial metabolites, including alpha-glucosidase inhibitors [34], and PG in this study. The results suggested that marine chitinous wastes should be preprocessed before application for fermentation. 

PG production by fermentation has been reported in numerous studies; however, almost all of them used commercial nutrient mediums [10,11,12,13,14,15] or agricultural products [6,16,17,18,19,20] for fermentation. Different from previous studies, we used a designed medium containing the low-cost material de-SSP for the cost-effective biosynthesis of PG. In addition, de-SSP represented a newly found potential source for cost-effective PG production by *S. marcescens* in this study. 

### 2.2. Establishment of the Process for de-SSP Bioprocessing into PG by S. marcescens on a Small Scale

We investigated the effect of different *S. marcescens* strains (1), different free protein sources (2), salt composition (3), and various parameters of cultivation on PG production (4) on small-scale fermentation (100 mL flask).

(1) PG production by different *S. marcescens* strains

Different bacterial strains of *S. marcescens* produce PG metabolites to different extents in the same conditions [31,33]. To determine the most active PG-producing strain to convert de-SSP into PG, a total of four strains of *S. marcescens* were examined for fermentation. The results (Table 1) showed that all the tested strains could produce high PG yields in the range of 3.562–4.015 mg/mL. Of these strains, *S. marcescens* TNU01 biosynthesized PG with a slightly higher yield than the other strains. Thus, this strain was chosen for further experiments.

(2) The influence of different free protein sources on PG production

**Figure 2:**
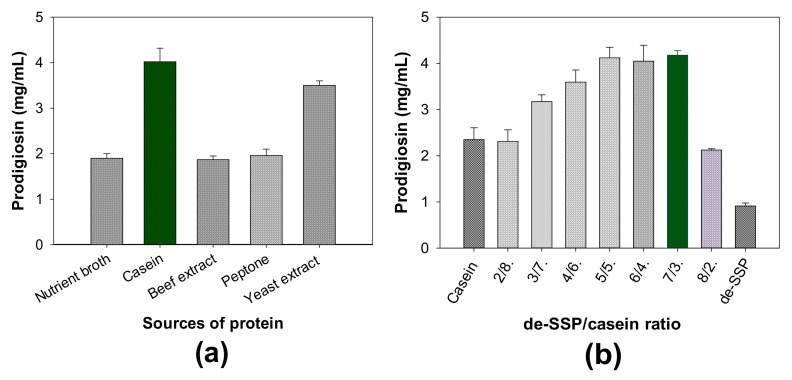
The influence of protein sources (**a**) and de-SSP/casein ratio (**b**) on PG productivity via fermentation by *S. marcescens* TNU01. Carbon/nitrogen sources in different proteins were combined with de-SSP at a ratio of 3.0/7.0 (**a**) and de-SSP was combined with casein in various ratios ranging from 2/8 to 8/2 (**b**) were used at the concentration of 1.60% in a liquid medium containing 0.03% K_2_HPO_4_ and 0.05% CaSO_4_. Oral casein and oral de-SSP were also fermented for comparison. The fermentation was performed for two days with no light, at 150 rpm and 25 °C. The error bars in the figures are standard errors (SE).

In some previous reports [31,33], the addition of free protein sources to the culture medium significantly enhanced PG biosynthesis by *S.* *marcescens.* Thus, to evaluate the effect of free protein sources on PG production, five sources of free protein were added to a medium containing de-SSP and fermented for two days by *S. marcescens* TNU01. Of these tested proteins, casein was found to be the most suitable protein source. The medium supplemented with casein reached the highest PG yield of 3.991 mg/mL and was thus used for further experiments to assess the most suitable ratio of de-SSP/casein. As shown in Figure 2b, the combination of de-SSP and casein at the ratios of 7/3, 6/4, and 5/5 gave a high PG yield of 4.05–4.18 mg/mL. To utilize shrimp wastes for cost-effective PG production, the de-SSP/casein at the ratio of 7/3 was chosen for further investigations. Oral casein and oral de-SSP were also fermented for comparison. As shown in Figure 2b, these two control mediums reached lower PG yields of 2.35 and 0.91 mg/mL, respectively, than that (4.18 mg/mL) of the mixture medium (de-SSP/casein = 7/3). 

Demineralized shrimp shell powder (de-SSP) and protein (casein) were mixed at the ratio of 7/3 and used as a C/N source for fermentation. A 1.6% C/N source was added to a liquid medium containing 0.03% K_2_HPO_4_ and 0.05% CaSO_4_. The fermentation was performed for two days at 25 °C in the dark at a shaking speed of 150 rpm.

(3) The influence of salt composition on PG production

**Figure 3:**
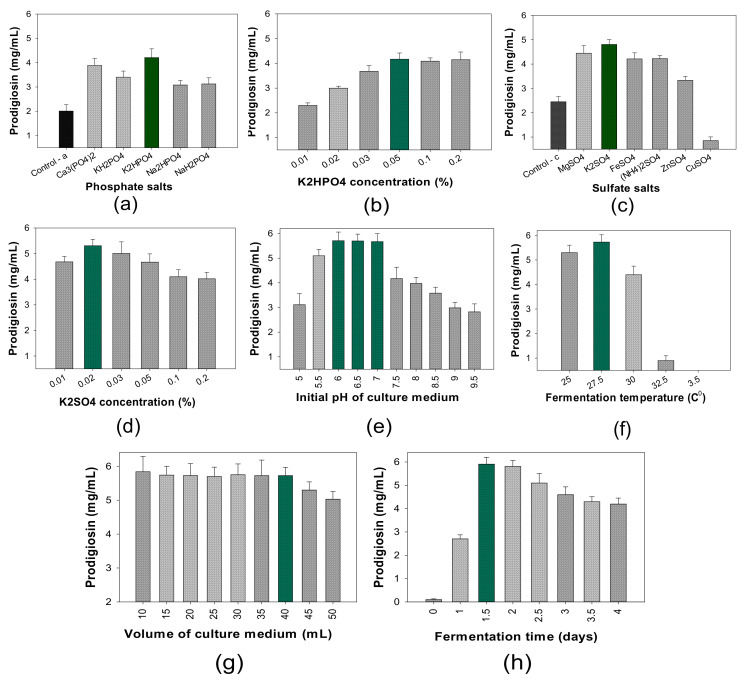
The influence of phosphate salt sources (**a**), KH_2_PO_4_ concentration (**b**), sulfate salt sources (**c**), K_2_SO_4_ concentration (**d**), pH of the liquid medium (**e**), temperature of fermentation (**f**), volume of the liquid medium (**g**), and time course for fermentation (**h**) on PG yield by *S. marcescens* TNU01 fermentation. The error bars in the figures are standard errors (SE).

A suitable composition of phosphate and sulfate salts has shown enhanced PG productivity by *S. marcescens* strains [31,32,33]. For more effective PG production in this study, various kinds of phosphate and sulfate salts were utilized for fermentation (Figure 2). The results indicated that, compared to other phosphate salts, the addition of K_2_HPO_4_ salt into the culture medium resulted in the highest PG synthesis by the TNU01 strain (Figure 3a). A control group (no addition of phosphate salt) was also investigated for comparison, and this control group showed the lowest PG yield compared to all experimental groups. In the next experiment (Figure 3b), the addition of K_2_HPO_4_ at a concentration of 0.05% was found to be the most effective on PG production by *S.* *marcescens* TNU01. Thus, K_2_HPO_4_ salt at its optimal concentration of 0.05% was further combined with various kinds of sulfate salts to screen the most effective sulfate salt on PG biosynthesis. Among the various examined sulfate salts, K_2_SO_4_ was found to be the best source of sulfate salt (Figure 3c) when added to the medium at a low concentration of 0.02% (Figure 3d). This was the first study to report the use of K_2_SO_4_ as a potential sulfate salt source for the significant enhancement of PG production by *S. marcescens.*

(4) The influence of cultivation parameters on PG production

To reach the highest PG yield produced by *S. marcescens* TNU01, some parameters of cultivation, including the initial pH, fermentation temperature, volume of the liquid medium, and time courses of fermentation were examined (Figure 3e–h). *S. marcescens* TNU01 produced the highest PG yield at an initial pH of 6–7 (Figure 3e) and culture temperature of 27.5 °C (Figure 3d). These results were similar to those reported in many previous studies [2,5,31,33,35,36,37,38,39]. These optimal factors (initial pH = 7 and culture temperature = 27.5 °C) were used in the next experiments to investigate the effect of culture volume and time course of fermentation. As shown in Figure 3g, PG was produced with a high yield when the culture liquid medium volumes were controlled in the range of 10–40 mL in a 100 mL flask. Regarding the large-scale harvesting of PG, the maximum culture medium volume of 40 mL in a 100 mL flask (culture medium/flask volume ratio = 4/10) was considered for further studies. This recorded result was similar to some of our previous works [2,31,33]. The fermentation time needed to harvest the maximum PG yield was also examined. The result shown in Figure 3 indicates that *S. marcescens* TNU01 produced PG with the highest yield cultivation time at 1.5–2 days. Although some factors (initial pH, culture temperature, volume of medium) for PG production by *S.* *marcescens* TNU01 were similar to those reported in many previous studies, this bacterial strain could produce the maximum yield of PG in a shorter fermentation time (36 h) relative to that (48–84 h) reported in some previous works [2,5,31,35,36,37,38,39]. 

Overall, *S. marcescens* TNU01 was found to produce the highest yield of PG when using the novel-designed medium containing a 1.60% source of C/N (de-SSP/casein = 7/3), 0.02% K_2_SO_4_, 0.05% K_2_HPO_4_, and a number of fermentation parameters, including an initial pH of 6–7, a medium culture volume of 40 mL, and culture temperature of 27.5 °C for 1.5 days. Notably, the PG yield was significantly enhanced after optimization of the culture conditions and showed an approximately 1.5—fold increase (3.98–5.91 mg/mL). The fermentation conditions and PG yield before and after optimization are mentioned in Table 2.

To date, various methods have been used for PG biosynthesis, including immobilized cultures, batch fermentation, continuous fermentation, and fermentation in bioreactor systems [2,33,40,41]. Of these, bioreactor cultures are suitable for industrial fermentation and the large-scale production of microbial metabolites such as PG. In addition, it has been suggested that the production of PG in a bioreactor system results in PG production on a large scale with higher productivity in a shorter cultivation time [33]. Thus, in this study, we preinvestigated PG production in a flask (small scale) and then scaled up PG production using a 15 L bioreactor system. 

### 2.3. Scale-Up of PG Production in a Bioreactor System and the Purification and Qualification of S. marcescens TNU01 PG

**Figure 4:**
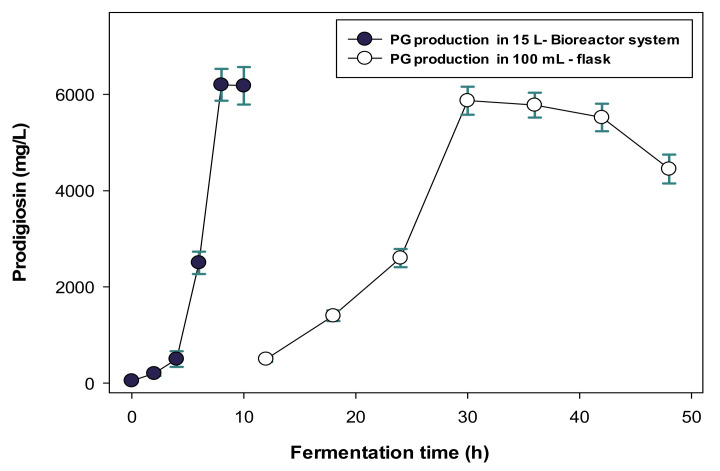
Production of PG in a 15 L bioreactor system and 100 mL flask for different periods of time. The error bars in the figures are standard errors (SE).

To scale up the PG biosynthesis, a bioreactor system with a full volume of 15 L was used for fermentation. The biosynthesis of PG on a small-scale in a 100 mL flask was also conducted for comparison. As shown in Figure 4, *S. marcescens* TNU01 produced the highest PG yield of 6200 mg/L after eight hours of fermentation, at which time the color of the culture broth appeared the reddest (Figure S1 presented in the Supplementary Materials). In contrast, this bacterium produced a lower yield of PG (5870 mg/L) over much longer time courses of fermentation (30 h) in a 100 mL flask.

To date, many studies have produced PG with a high yield. However, in nearly all of these previous works, PG was produced on a small scale, in flasks, using commercial nutrients as a C/N source for fermentation [1,2]. To scale up PG production, bioreactor systems have been utilized for fermentation in some previous studies [35,36,37,38]. As summarized in Table 3, many different sizes of bioreactors, such as 1.5, 5, 7, 10, 15, and 100 L, with true working volumes of 0.935, 2.75, 6.5, 3, 4.5, and 50 L, respectively, were examined for PG production in mass, and the reported PG yield was in the range of 521.64–872 mg/L [40,41,42,43,44]. However, all these previous reports used commercial nutrients as C/N for fermentation. Unlike them, we established the bioconversion of MCWs for the production of PG and successfully approached PG production on a large scale of 3 L [2] and 4.5 L [33] with a high PG yield of 3450 and 5100 mg/mL, respectively. In this study, a 15 L bioreactor system with a working volume of 5.0 L was used for fermentation to produce a high yield of PG (6200 mg/L). Notably, the fermentation time needed to produce the highest PG yield in the bioreactor system in this study was significantly shorter (8.0 h) than most previous reports (12–65 h). 

The red compound was extracted and isolated from the fermented broth in the bioreactor system (Figure S2a) following the assay presented in detail in our previous study [31]. In brief, this process had several steps, including separation of different liquid layers using ethyl acetate to obtain an extract rich in PG (Figure S2b), fractionation of this crude extract by a column loaded with silica gel (Figure S2c), and the final separation and isolation of the red compound via thin-layer chromatography (Figure S2d). The red compound was confirmed as PG by analysis of its HPLC profile, mass, and UV/vis spectra. As shown in Figure S3, both the PGs obtained from our earlier report [33] (Figure S3a) and the red compound purified in this study (Figure S3b) appeared as a single peak at the approximate same retention time in the range of 12.283–12.40 min. In addition, this red compound had a molecular weight and max UV/vis of 323.2063 g/mol (Figure S4) and 535 nm (Figure S5), respectively, which were similar to the mass and optimal UV/vis absorption of PG [2,33,45]. Thus, the isolated red compound was confirmed as PG.

### 2.4. Evaluation of the Biological Effects of Prodigiosin

To date, this pigment compound has been shown to carry out numerous biological activities [1]. Various studies have reported the potential inhibitory effect of PG against numerous cancerous cell lines [1]. To confirm the PG produced in this study was an active anticancer compound, its bioactivity on several cancerous cell lines was tested. As shown in Table 4, PG exerted its potent anticancer effect on a number of cell lines, such as A549, MCF-7, WiDr, and HepG2, which all recorded low IC50 values of 0.07, 0.05, 0.22, and 0.06, respectively. The commercial anticancer compound mitomycin was also tested, and its IC50 values were 0.15, 0.11, 0.13, and 0.14, respectively. Thus, PG demonstrated lower inhibition against WiDr but much higher inhibitory activity against A549, MCF-7, and Hep G2, compared to that by mitomycin. 

Antioxidants have been proven to protect DNA, proteins, and lipids from damage due to free radicals. Thus, antioxidant compounds may help to reduce and prevent cells from a vast array of diseases [2,46]. In this study, assays of the DPPH and ABTS radical scavenging effect were used to detect antioxidant activity. To compare the antioxidant effects, a standard antioxidant agent (α-tocopherol) was also tested under the same conditions. As shown in Table 5, the antioxidant compound α-tocopherol showed effective DPPH and ABTS radical scavenging effects with the very low IC50 values of 24.3 and 12.7115 µg/mL, respectively, while PG demonstrated moderate antioxidant effects, with IC50 inhibition values of 235 µg/mL for DPPH and 115 µg/mL for the ABTS radical scavenging effects. Many studies have reported that GP shows DPPH radical scavenging activity with a potent maximum inhibition of 86% [47], 98% [2], 99% [48], and 96% [33]. PG also shows ABTS radical scavenging properties [33], with a maximum inhibition of 98.3% and a moderate IC50 value of 1.25 mg/mL. However, the ABTS radical scavenging activity of PG has been rarely reported [33]; thus, the data on the ABTS radical scavenging capacity in this study could contribute to the available antioxidant data of PG.

**Figure 5:**
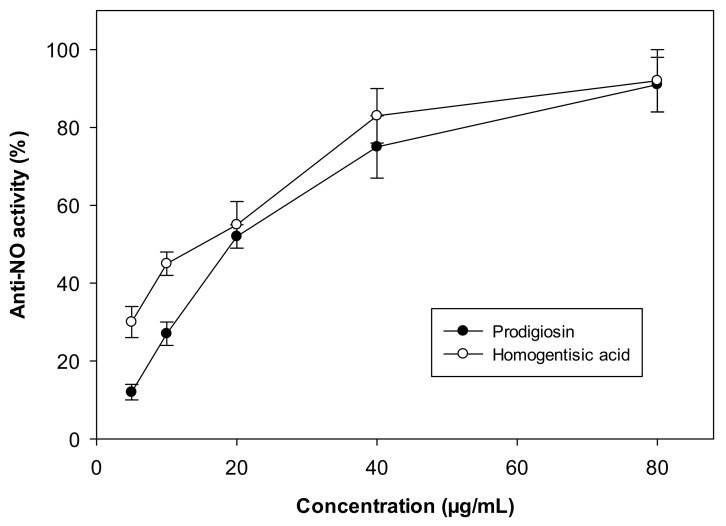
The anti-NO activity of prodigiosin (PG) produced via fermentation in this study. The error bars in the figures are standard errors (SE).

The anti-nitric oxide effect of PG was also examined (anti-NO, an indicator of pro-inflammatory properties, which is related to some disorders such as rheumatoid arthritis, chronic hepatitis, and pulmonary fibrosis) [30,49,50,51]. In this report, LPS-stimulated-RAW264.7 cells were used to assess the anti-NO activity of PG, and homogentisic acid was used as the standard for the anti-NO assay. As shown in Figure 5, PG showed potent anti-NO properties with a maximum inhibition of 91%, which was comparable to that of homogentisic acid (92%) at 80 µg/mL. To clarify the results, the anti-NO activity was also recorded as IC50 values. PG showed a low IC50 value of 19.1 µg/mL, and this activity was comparable to that of homogentisic acid (IC50 = 15.9 µg/mL). The biological activities of PG have been increasingly reported, especially anticancer activities. However, few data on the anti-NO activity of PG are available. In a previous report, PG was reported to show pro-inflammatory activities via an in silico assay [52]. Thus, this study supported the in vitro study data to confirm the novel potential anti-NO effect of PG on LPS-stimulated-RAW264.7 cells.

## 3. Materials and Methods

### 3.1. Materials

The *S. marcescens* strains were obtained from previous works, such as *S. marcescens* TKU011 [5], *S. marcescens* TNU01, *S. marcescens* TNU02 [31], and *S. marcescens* CC17 [53]. The marine chitinous wastes (MCWs), including shrimp shells, shrimp heads, crab shells, and squid pens were provided by Shin-Ma Frozen Food Co. (I-Lan, Taiwan), and demineralization of the MCWs was performed following the method detailed in an earlier study [54]. The cancer cell lines MCF-7, A549, Hep G2, and WiDr were purchased from the Bioresources Collection and Research Centre (Hsinchu, Taiwan). 

### 3.2. Methods

#### Study of Bioproduction of Prodigiosin via Bacterial Fermentation

The Influence of Different S. marcescens Strains on PG Production

A total of four Serratia marcescens strains, including TKU011, TNU01, TNU02, and CC17, were used for PG production. Demineralized shrimp shell powder (de-SSP) and protein (casein) were mixed at the ratio of 7/3 (de-SSP/casein) and utilized as a C/N source for fermentation. The C/N source (1.6%) was added to a liquid medium containing 0.03% K_2_HPO_4_ and 0.05% CaSO_4_. Fermentation was performed for two days at 25 °C in the dark at a shaking speed of 150 rpm (*).

The Effect of Different Free Protein Sources on PG Production

Different proteins (nutrient broth, casein, beef extract, peptone, and yeast extract) were combined with de-SSP at the ratio of 3/7 and used as the C/N source for fermentation. The C/N source (1.6%) was added to a liquid medium containing 0.03% K_2_HPO_4_ and 0.05% CaSO_4_. The fermentation was carried out as mentioned above (*). Casein was screened as the most suitable free protein source. Therefore, this protein was further investigated for its suitable concentration to the medium. Various ratios of de-SSP combined with casein at a ratio of 2/8–8/2 were used at a concentration of 1.6% in a liquid medium containing 0.03% K_2_HPO_4_ and 0.05% CaSO_4_, and fermentation was performed in the same condition as mentioned above (*).

The Effect of Phosphate Type and Concentration on PG Production

Several types of phosphates, such as Ca_3_(PO_4_)_2_, KH_2_PO_4_, K_2_HPO_4_, Na_2_HPO_4_, and NaH_2_PO_4_, were tested. The liquid medium contained a 1.6% source of C/N, 0.03% phosphate salt, and 0.05% CaSO_4_, and fermentation was performed in the same condition as mentioned above (*). K_2_HPO_4_ demonstrated the most enhancing effect on PG yield; thus, it was further added to the culture medium at the concentration range of 0.01, 0.02, 0.03, 0.05, 0.1, and 0.2%, and fermentation was performed in the same condition as above (*) to explore the optimal K_2_HPO_4_ concentration.

The Effect of Sulfate Type and Concentration on PG Production

Various types of sulfates of (NH_4_)_2_SO_4_, K_2_SO_4_, FeSO_4_, MgSO_4_, ZnSO_4_, and CaSO_4_, were examined for their effect on PG productivity. The culture broth contained a 1.6% source of C/N, 0.03% K_2_HPO_4_ salt, and 0.05% sulfate salt, and fermentation was performed in the same condition as mentioned above (*). K_2_SO_4_ demonstrated the most enhancing effect on PG yield; thus, it was further added to the culture medium at the concentration range of 0.01, 0.02, 0.03, 0.05, 0.1, and 0.2%, and fermentation was performed in the same condition as mentioned above (*) to explore the optimal K_2_SO_4_ concentration.

The Effect of Cultivation Parameters on PG Production

To achieve the highest PG yield, a liquid medium containing a 1.6% source of C/N (de-SSP/casein = 7/3), 0.02% K_2_SO_4_ and 0.05% K_2_HPO_4_ was fermented by *S. marcescens* TNU01 under different conditions but with some parameters, including the initial pH of the liquid medium (a pH range of 5–9.5), fermentation temperature (25, 27.5, 30, 32.5. and 35 °C), volume of the liquid medium (10, 15, 20, 25, 30, 35, 40, 45, and 50 mL in a 100 mL flask), and time courses for fermentation (0–4 days) were examined. All the following experiments were designed according to the optimal parameters achieved from previous experiments.

Scale-Up Production of PG in the Bioreactor System

A 15 L BioFlo/CelliGen 115 bioreactor system (Eppendorf North America, Enfield, CT, USA) was used for fermentation. The optimal culture conditions explored from all the above-mentioned experimental results were applied for the mass production of PG in a bioreactor. A total of 500 microliters of bacterial seed were cultured in a 1 L flask at 27 °C for 1.50 days and then injected into the fermenter system containing 4.5 L of medium. This medium contained a 1.6% source of C/N (de-SSP/casein = 7/3), 0.02% K_2_SO_4_, and 0.05% K_2_HPO_4_ with an initial pH of 6–7, and fermentation was conducted at 27.5 °C in the dark. Sampling and detection of the PG yield were performed every two hours (from 0 to 10 h of fermentation).

### 3.3. Qualification, Extraction, and Purification of Prodigiosin 

PG was qualified according to the method described in a previous study [32]. A cultured broth (0.25 mL) was mixed with 2 mL methanol and 0.25 mL of 2.0% AlK(SO_4_)_2_‧12H_2_O, and the mixture was then centrifuged at 1400× *g* for five minutes. This supernatant (0.5 mL) was added to a flask containing 4.50 mL acidic methanol adjusted by 0.5 HCl, and the optical density (OD_535nm_) of this final solution was measured. The PG compound previously isolated in our earlier work [33] was used to establish the standard equation for converting the OD_535nm_ value into the PG concentration. 

The extraction and purification of PG were carried out according to the method presented in a previous report [33]. The cultured broth in the bioreactor system at optimal conditions was centrifuged for 10 min at 10,000× *g*. The supernatant was then collected. Ethyl acetate (EA) was added to this supernatant at an equal volume and the mixture was kept for three hours in a glass funnel, with shaking performed every 30 min. The EA layer was then collected. The residue in the supernatant was further mixed with EA twice for the complete extraction of PG. All the EA layers were mixed together for evaporation in a rotary evaporator (IKA, Germany) at 55 °C and then dried to a powder (crude PG). This crude pigment was separated by loading it onto a column of silica gel (Geduran^®^ Si 60, size: 0.040–0.063 mm). The solvent system contained methanol and chloroform with a gradient from 0/10 to 2/8 (*v*/*v*) for the elution of PG, and the pigment was finally isolated after separation by thin-layer chromatography plated with the solvent system of methanol/chloroform (1/9). The target compound in the red band was completely cut out into small pieces, and methanol was utilized for the extraction of the red pigment compound. The methanol-rich PG was dried in a rotary evaporator (IKA, Germany) at 55 °C and then dried to powder (crude PG). This purified compound was used to assess its bioactivities as well as examine its mass, UV/vis spectra, and HPLC. 

### 3.4. Detection of the Bioactivities of Prodigiosin

The PG purified in this study was used for the evaluation of certain biological effects, including anticancer, antioxidant, and anti-NO activities. ABTS and DPPH assays were used for the detection of antioxidant activities according to the previous studies [48,49], respectively. The anticancer properties were examined according to the method reported in our earlier work [55]. The anti-NO effect was also tested according to the assay presented in our earlier report [50]. 

## 4. Conclusions 

Prodigiosin (PG) has received much attention due to its numerous beneficial biological activities. The potential applications of PG have led to a dramatic increase in the investigation of PG biosynthesis. However, in almost all previous reports, commercial nutrient mediums were used as C/N sources for fermentation, and PG was produced small-scale in flasks. This study utilized shrimp wastes (low-cost materials) as the source of C/N for the production of PG by bacterial fermentation and used a bioreactor system for scale-up PG production. The PG yield produced by *S. marcescens* TNU01 in the bioreactor system in this work reached a higher yield (6200 mg/L) than that of some previous reports (521.64–872 mg/L). Notably, the fermentation time for max PG yield production in this study was also significantly shorter (8.0 h) than the reported studies (12–65 h). The red pigment purified from the cultured medium was confirmed as PG via analyzing its HPLC profile, mass, and UV/vis spectra. The purified PG showed effective anticancer activities, moderated antioxidant activities, and anti-NO effects. PG has been reported to possess various biological effects, especially anticancer properties. However, few data on the anti-NO activity of PG are available. The results obtained in this study suggested that shrimp wastes are potentially used for the cost-effective production of bioactive PG in mass.

## Figures and Tables

**Table 1:** PG production by different *S. marcescens* strains.

*No.*	*S. marcescens* Strains	PG Concentration in Culture Broths (mg/mL)
*1*	TKU011	3.832 ± 0.142
*2*	TNU01	4.015 ± 0.161
*3*	TNU02	3.721 ± 0.177
*4*	CC17	3.562 ± 0.145
	Control (no bacterium)	-

**Table 2:** The fermentation conditions and PG yield before and after optimization.

Factors	Initial Experiment	Optimal Conditions
*S. marcescens* strain	TNU02	TNU01
C/N source	de-SSP/casein = 7/3	de-SSP/casein = 7/3
Salts compositions	0.03% K_2_HPO_4_ and 0.05% CaSO_4_	0.02% K_2_SO_4_ and 0.05% K_2_HPO_4_
Initial pH of medium	6.15	7.0
Fermentation temperature (°C)	27	27
Ratio volume of medium/flask	3/10	4/10
Time course of fermentation (day)	2	1.5
PG yield (mg/mL)	3.98	5.61

**Table 3:** Scale-up of PG production in bioreactors from different studies.

Strain	C/N Sources	Scale Production	PG Yield	Culture Time (h)	Ref.
*S. marcescens* TNU01	1.12% de-SSP/0.48% casein	5/15 L	6200 mg/L	8	This study
*S. marcescens* TNU01	1.75% SPP	3/10 L	3450 mg/L	12	[2]
*S. marcescens* TNU02	1.12% de-CSP /0.48% casein	4.5/15 L	5100 mg/L	8	[33]
*S. marcescens* 02	1.0% glycerol, 1.0% tryptone, 1.0% extract of yeast	2.75/5 L	583 mg/L	20	[40]
*S. marcescens*	0.865% sucrose/0.662% peptone	6.5/7 L	594.88mg /L	52	[42]
*S. marcescens* BS 303 (ATCC^®^ 13880™)	3.0% glycerol/1.05% casein peptone	0.935/1.5 L	872mg/L	65	[43]
*Chryseobacterium artocarpi* CECT 849	1.125% Lactose and 0.6% l-tryptophan.	50/100 L	521.64 mg/L	24	[44]

**Table 4:** The anticancer effect of PG.

	Inhibitory Activity against Cancer Cell Lines (IC50 Value, µg/mL)			
	A549	MCF-7	WiDr	Hep G2
PG	0.07 ± 0.015	0.05 ± 0.009	0.22 ± 0.02	0.06 ± 0.015
Mitomycin C	0.15 ± 0.011	0.11 ± 0.009	0.13 ± 0.012	0.14 ± 0.021

**Table 5:** The radical scavenging activity of PG.

	DPPH Assay		ABTS Assay	
	Max Inhibition Value (%)	IC50 Value (µg/mL)	Max Inhibition Value (%)	IC50 Value (µg/mL)
PG	99 ± 1.67	235 ± 19.32	100 ± 1.98	115 ± 10.12
α-tocopherol	100 ± 2.21	24.3 ± 1.62	97 ± 1.81	12.7 ± 1.02

The max inhibition (%) of the samples was detected at 8 mg/mL (PG) and 50 µg/mL (α-tocopherol).

## Data Availability

Data available on request.

## Supplementary Materials

The production process in a 15 L bioreactor system, the extraction and purification, the HPLC profiles, the HREIMS, and the UV/vis spectrum of the purified prodigiosin (PG) produced via fermentation in this study are found in the supplementary material (Figures S1–S5).

## Sample Availability

Samples of the compound are available from the authors.
