# Supplementary material for: Bioprocessing of Marine Chitinous Wastes for the Production of Bioactive Prodigiosin
Source: Molecules. 2021 May 24;26(11):3138. doi: 10.3390/molecules26113138 (PMC8197340; doi:10.3390/molecules26113138)
Supplement: Supplementary file 1 [file molecules-26-03138-s001.zip › molecules-1178508-supplementary.pdf]

Supplementary materials

Article: **"Bio-processing of Marine Chitinous Wastes for the Production of Bioactive Prodigiosin"**

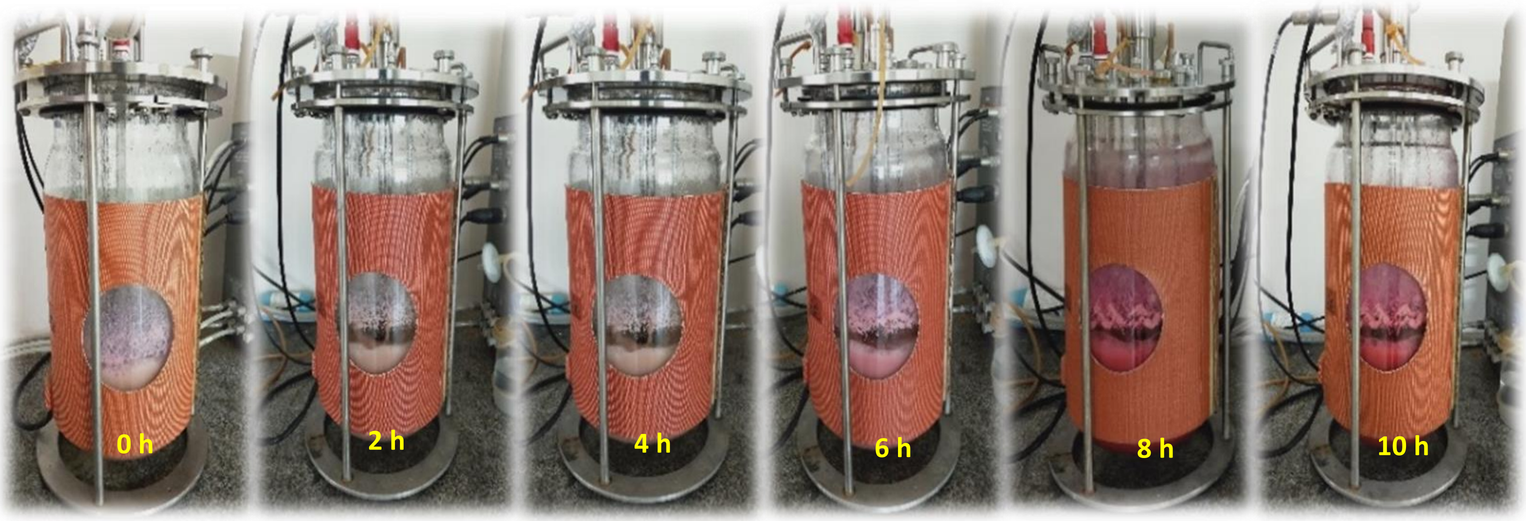

Figure S1. Production of prodigiosin (PG) in a 15 L bioreactor system. The red color increased significantly at 8 h of fermentation.

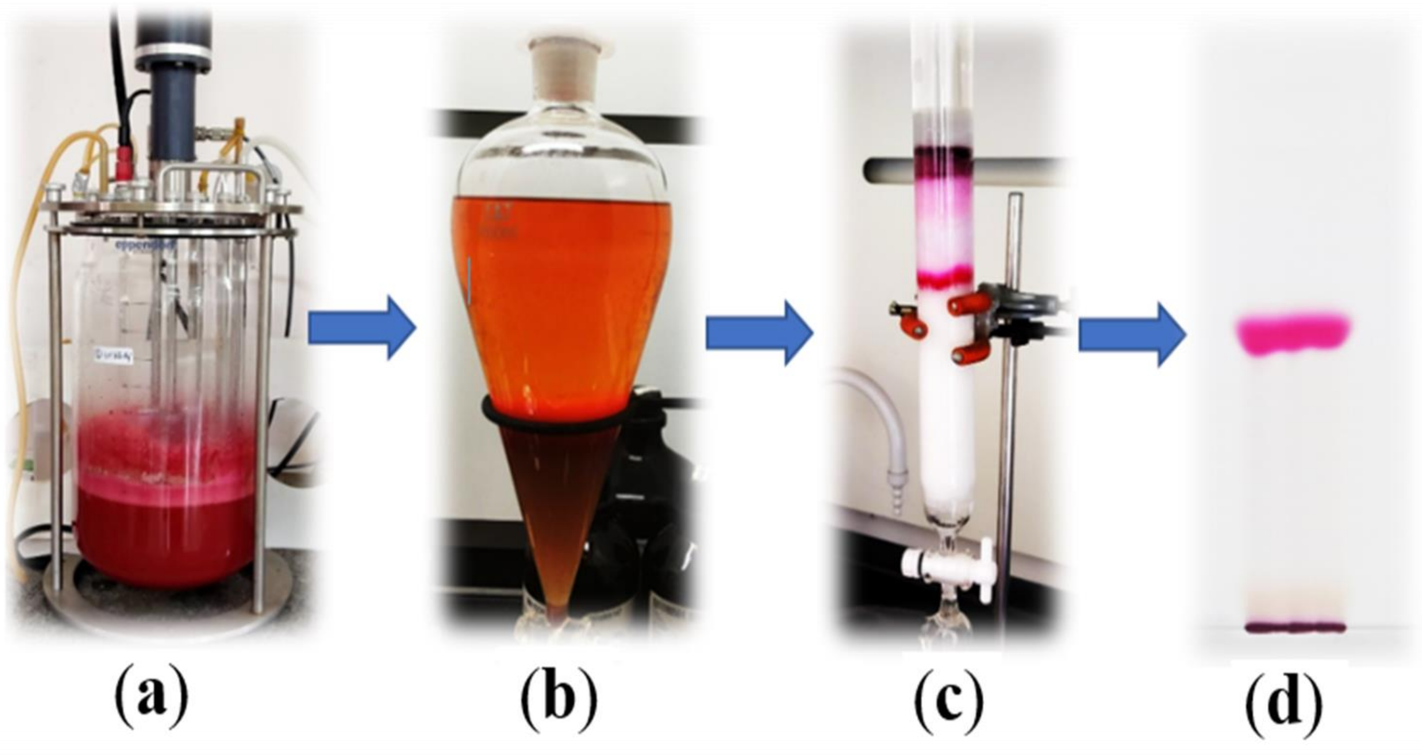

Figure S2. The extraction and purification of prodigiosin (PG). The red compound in the fermented medium in the 15-L bioreactor system (**a**) was purified via separation by ethyl acetate (**b**), fractionated in a silica gel column (**c**), and finally extracted using thin layer chromatography (**d**).

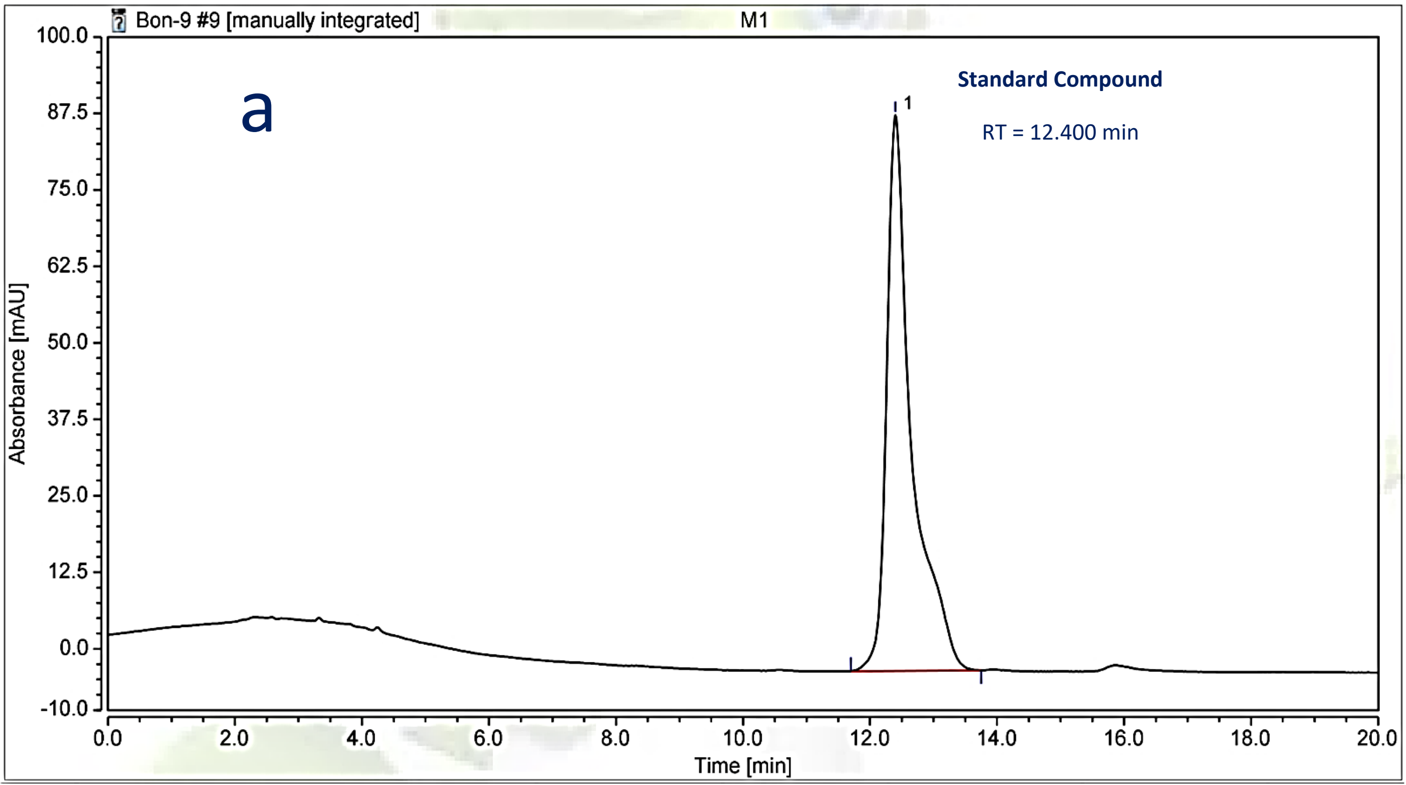

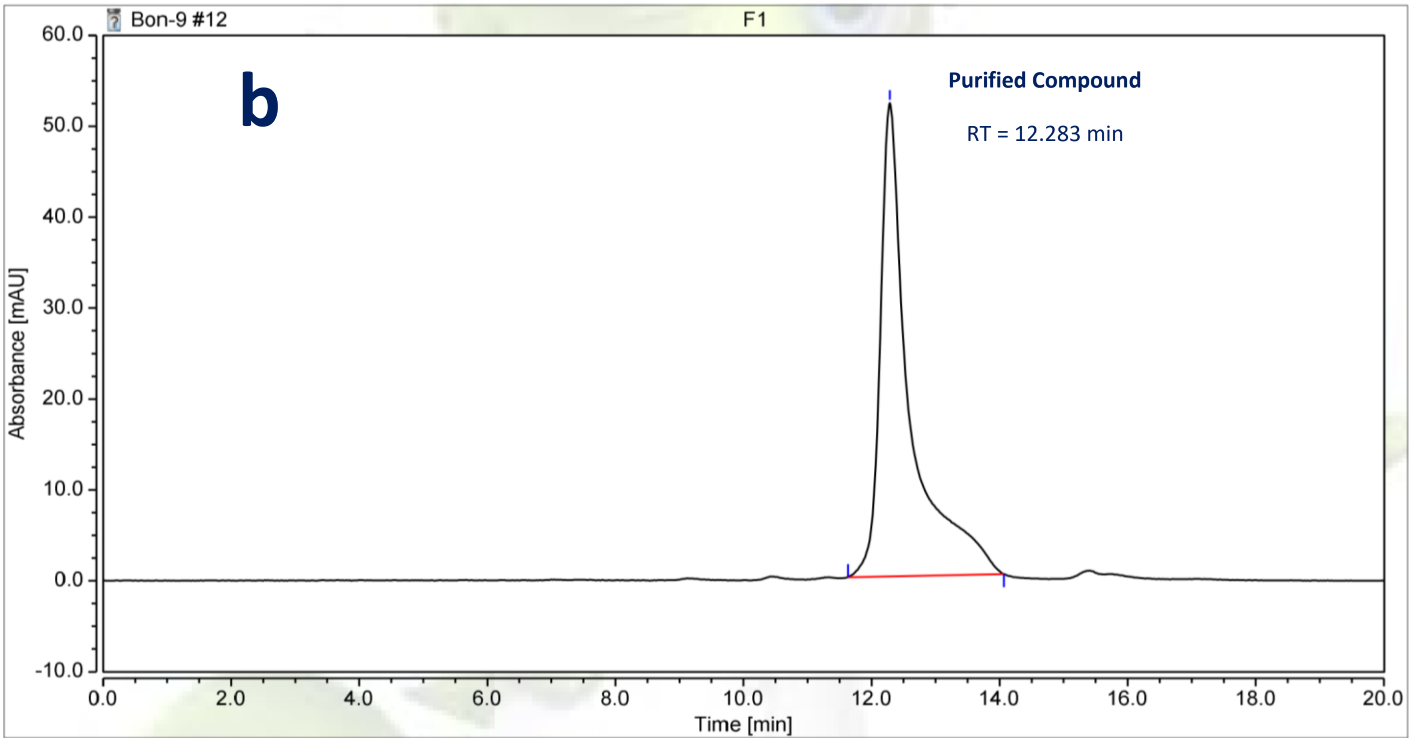

Figure S3. The HPLC profiles of prodigiosin (PG) purified in a previous study [33] (**a**) and prodigiosin purified in this study (**b**). High-performance liquid chromatography was utilized for the analysis. The samples were dissolved in methanol (MeOH) with a concentration of 1 mg/mL, and 3 μL was injected into the HPLC system. The sample was separated in a C18 column and detected at 535 nm under the following conditions: mobile phase 70% MeOH in water adjusted at pH 3.0 using 10 mM ammonium acetate with a stable flow rate of 0.8 mL, at a column temperature of 30°C for 20 min.

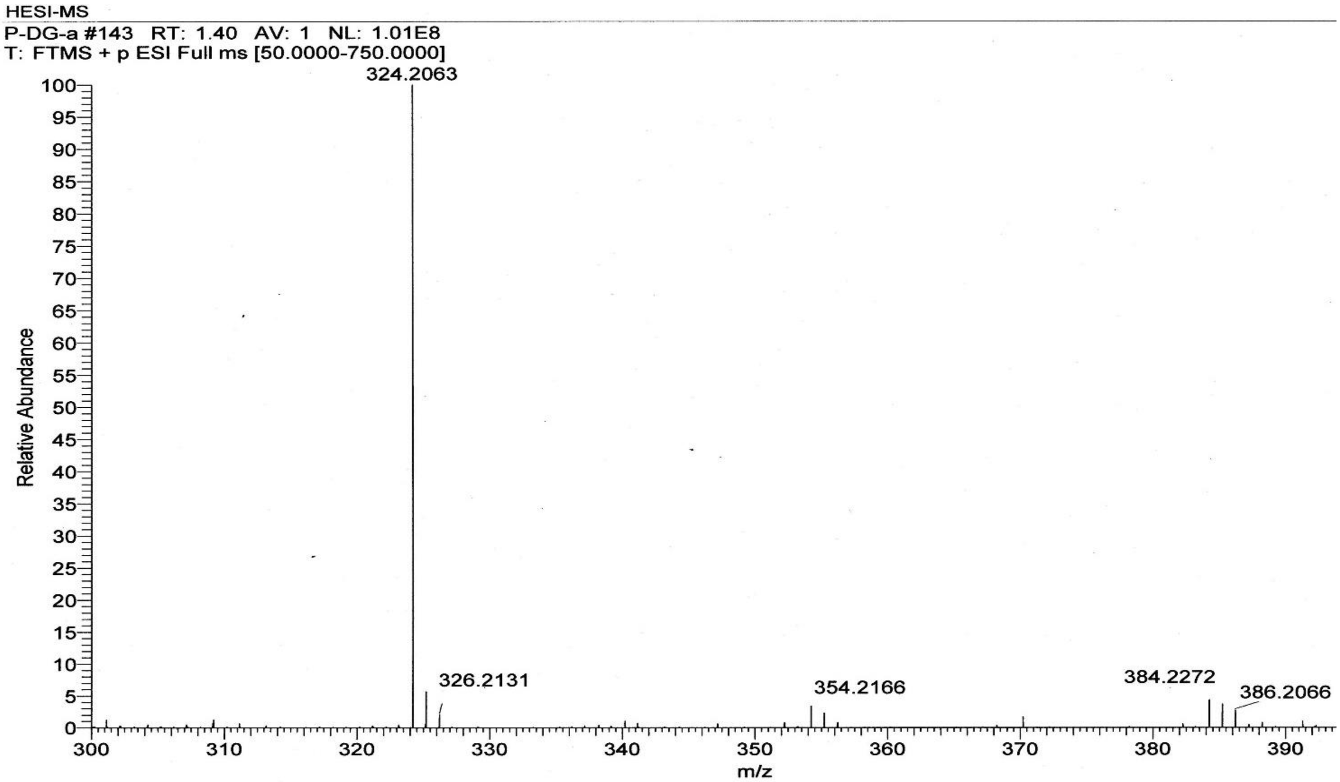

Figure S4. The HREIMS of the purified prodigiosin (PG) produced via fermentation in this study. [M+1] =324.2068.

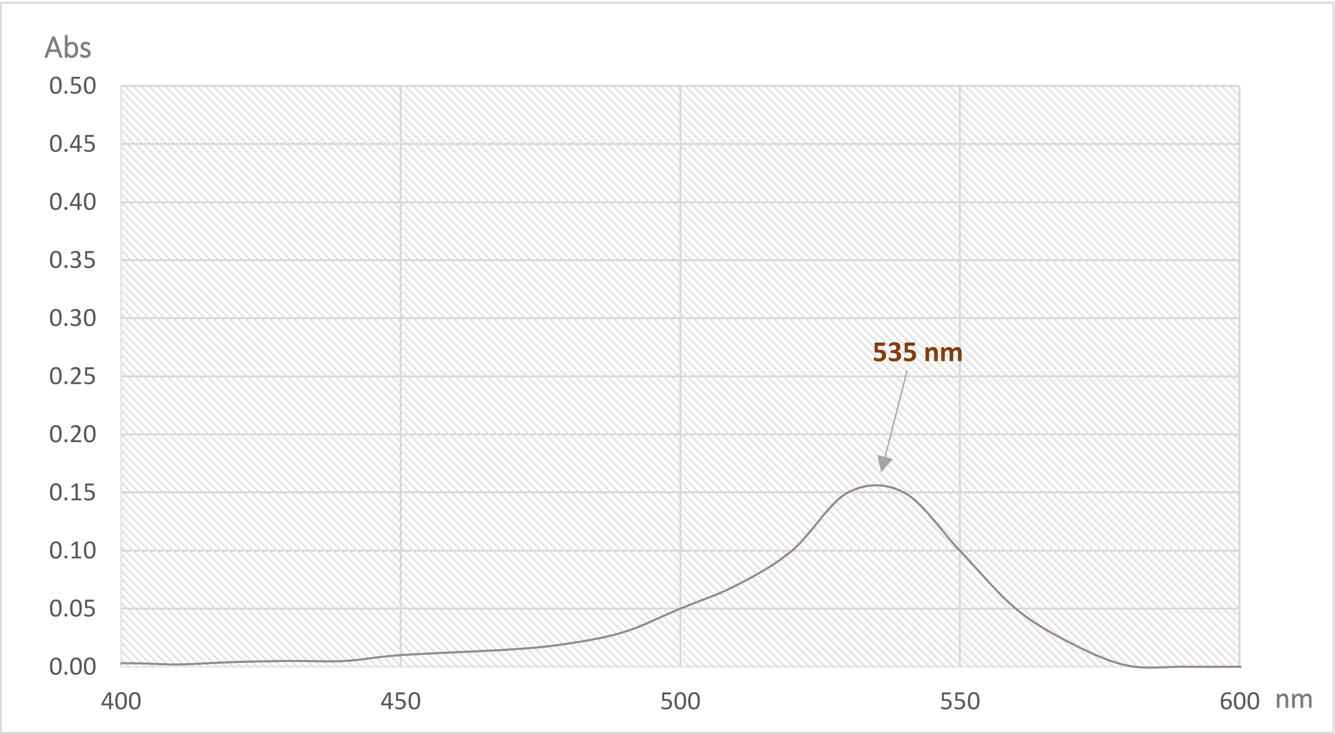

Figure S5. The UV/vis spectrum of the purified prodigiosin (PG) produced via fermentation in this study.
